# A tug-of-war between severe acute respiratory syndrome coronavirus 2 and host antiviral defence: lessons from other pathogenic viruses

**DOI:** 10.1080/22221751.2020.1736644

**Published:** 2020-03-14

**Authors:** Sin-Yee Fung, Kit-San Yuen, Zi-Wei Ye, Chi-Ping Chan, Dong-Yan Jin

**Affiliations:** a School of Biomedical Sciences, The University of Hong Kong, Pokfulam, Hong Kong; b Department of Microbiology, The University of Hong Kong, Pokfulam, Hong Kong

**Keywords:** Coronavirus, SARS-CoV, SARS-CoV-2, COVID-19, 2019 novel coronavirus, host antiviral response, type I interferon

## Abstract

World Health Organization has declared the ongoing outbreak of coronavirus disease 2019 (COVID-19) a Public Health Emergency of International Concern. The virus was named severe acute respiratory syndrome coronavirus 2 (SARS-CoV-2) by the International Committee on Taxonomy of Viruses. Human infection with SARS-CoV-2 leads to a wide range of clinical manifestations ranging from asymptomatic, mild, moderate to severe. The severe cases present with pneumonia, which can progress to acute respiratory distress syndrome. The outbreak provides an opportunity for real-time tracking of an animal coronavirus that has just crossed species barrier to infect humans. The outcome of SARS-CoV-2 infection is largely determined by virus-host interaction. Here, we review the discovery, zoonotic origin, animal hosts, transmissibility and pathogenicity of SARS-CoV-2 in relation to its interplay with host antiviral defense. A comparison with SARS-CoV, Middle East respiratory syndrome coronavirus, community-acquired human coronaviruses and other pathogenic viruses including human immunodeficiency viruses is made. We summarize current understanding of the induction of a proinflammatory cytokine storm by other highly pathogenic human coronaviruses, their adaptation to humans and their usurpation of the cell death programmes. Important questions concerning the interaction between SARS-CoV-2 and host antiviral defence, including asymptomatic and presymptomatic virus shedding, are also discussed.

Coronaviruses (CoVs) are found in various animals including aves and mammals. They can be divided into four genera named *Alphacoronavirus, Betacoronavirus, Gammacoronavirus*, and *Deltacoronavirus* [1]. The 2019 novel CoV (SARS-CoV-2) is the newest addition to human CoVs (HCoVs) that also include 229E, OC43, HKU1, NL63, severe acute respiratory syndrome (SARS) CoV, and Middle East respiratory syndrome (MERS) CoV. Whereas 229E and NL63 belong to *Alphacoronavirus*, others are members in the genus of *Betacoronavirus*. All of them are positive-stranded RNA viruses containing a polycistronic genome of ∼30 kb in size, coding for multiple non-structural proteins (ORF1a and ORF1b, processed into multiple nsp proteins) at the 5′-end plus multiple structural (S, E, M, and N) and lineage-specific accessory proteins (such as ORF3a, ORF3b, ORF6, ORF7a, ORF7b, ORF8a, ORF8b, and ORF9b in SARS-CoV) at the 3′-end (Figure 1). SARS-CoV and MERS-CoV are highly pathogenic and can cause severe diseases presented as acute respiratory distress syndrome (ARDS). Although the other four community-acquired HCoVs are a common cause of common cold only, they are thought to cause pandemics and major outbreaks of probably more severe respiratory diseases when they initially crossed species barriers to infect humans decades and centuries ago. All seven HCoVs have a zoonotic origin from bats, rodents, or domestic animals. Their reservoir hosts are selected through evolution. As a result of this selection and mutual adaptation for a long period of time, they usually become non-pathogenic or cause very mild diseases in their native reservoir hosts. However, when an animal CoV such as SARS-CoV-2 enters a new host such as humans, the severity of the disease is significantly increased at the start of a new round of adaptation. The outcome of infection is governed largely by the interplay between virus and host antiviral defence. Through years of co-evolution, this tug-of-war ultimately reaches a tie or a balance under which virus and host co-exist peacefully or even in mutual benefit. Understanding the host restriction factors and the viral countermeasures will shed significant new light on viral pathogenesis and antiviral development. Although it remains to be elucidated how SARS-CoV-2 interacts with host antiviral immunity, lessons can be learned from other HCoVs and human pathogenic viruses in other families including the human immunodeficiency viruses (HIVs). In this review, we focus on some of the key questions surrounding SARS-CoV-2 and its interplay with the host.

**Figure 1.**
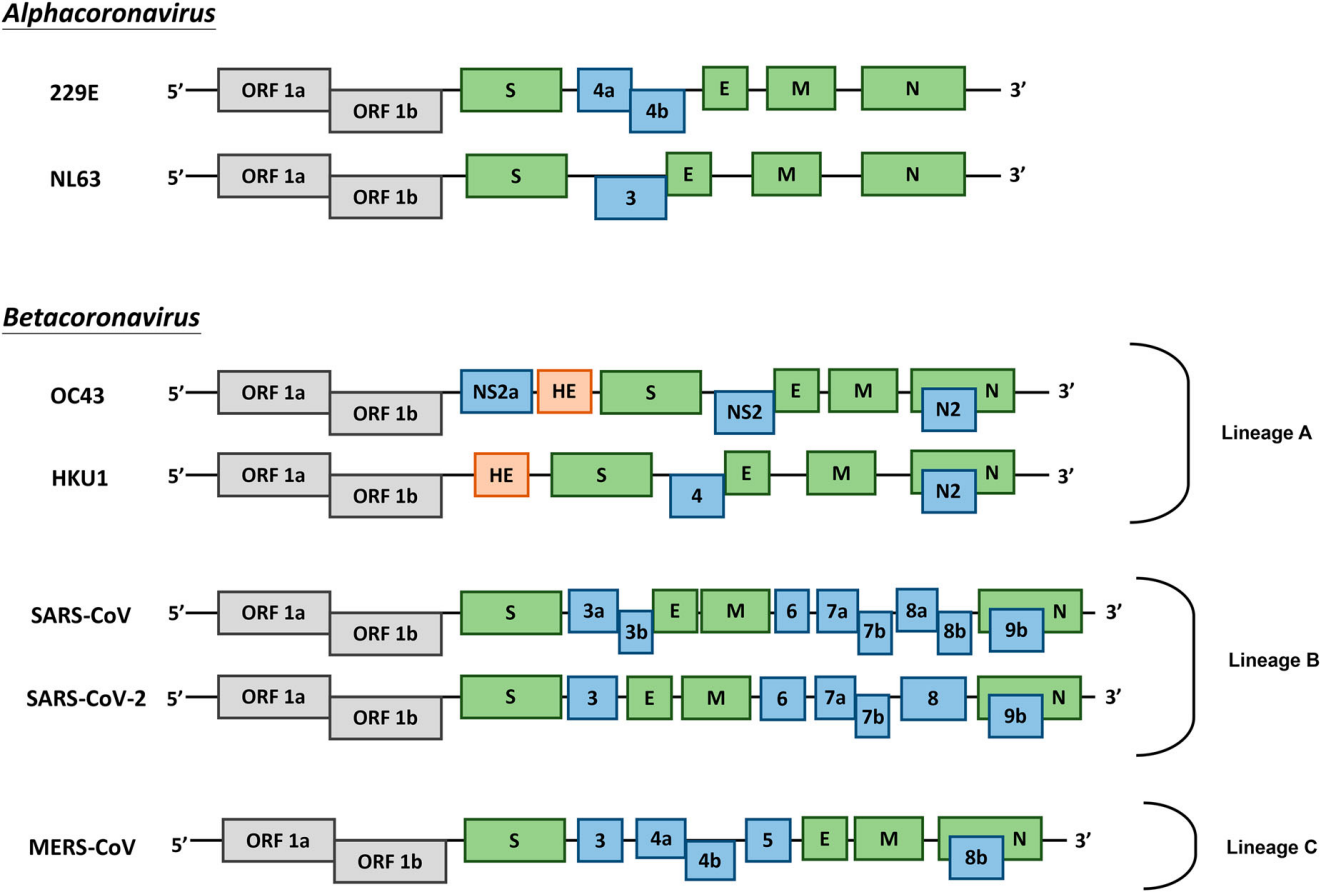
Genome organization of HCoVs. Schematic diagram of seven known HCoVs is shown (not in scale). The genes encoding structural proteins spike (S), envelope (E), membrane (M), and nucleocapsid (N) are in green. The gene encoding haemagglutinin-esterase (HE) in lineage A of betacoronaviruses is in orange. The genes encoding accessory proteins are in blue.

## Discovery of HCoVs

Avian infectious bronchitis virus was the first CoV isolated in 1930. Different CoVs were subsequently isolated in infected rodents and domestic animals, including mouse, pig, cow, turkey, cats, and dogs. CoVs were once believed not to cause human disease, but this was changed after the successful isolation of HCoV strain B814 from the clinical specimen of patients with common cold by serial passage of inoculum in tracheal organ culture in 1962 [2]. In the 1960s, several novel HCoVs were described but no further characterization was performed in most cases [3,4]. 229E and OC43 were known as causative agents of the common cold and upper respiratory tract infection, accounting for up to 30% of cases with common cold [4]. 229E is a prototype strain isolated using tracheal organ culture. OC43 was isolated from organ culture and subsequent serial passage in the brain of suckling mice. The clinical features of 229E and OC43 infection were characterized in human volunteer study [4]. In most cases, natural infection with HCoVs results in mild common cold-like symptoms. Severe lower respiratory tract infection develops only in immunocompromised patients [5]. Apart from a respiratory infection, 229E and OC43 were suspected to infect the central nervous system (CNS) as mRNA and CoV-like particles were detected in CNS samples of patients with multiple sclerosis. This claim was further supported by the susceptibility of human neural primary culture to 229E and OC43 [6]. However, the influence of 229E and OC43 on the development and progression of multiple sclerosis awaits further investigations.

In the pre-SARS era, it was generally accepted that HCoVs cause mild respiratory disease only. This concept was changed after the emergence of SARS-CoV. The first reported case of SARS-CoV infection was retrospectively dated back to November 2002 in Guangdong Province of China. In the subsequent seven months, the SARS epidemic resulted in over 8000 reported cases in 37 countries with a case fatality of 9.6% [7]. The superspreading events in SARS-CoV transmission caused fears in the society. Although the exact cause of superspreading remains to be understood, the host but not the virus only is thought to play a key role in the release of large amounts of virions in superspreading. In this regard, the use of immunosuppressive agents such as high-dose steroid in an early phase of viral infection as a treatment modality might boost viral replication leading to the shedding of large amounts of virus. Likewise, the immunocompromised status of the superspreader could have the same effect. In addition, mutation of virus susceptibility genes encoding restriction factors implicated in host antiviral defence would also result in the shedding of extraordinarily large quantities of the virus [8]. In other words, compromising host antiviral defence or decoupling host antiviral immune response from viral replication might allow or facilitate superspreading.

SARS-CoV was isolated and identified as the causative agent of SARS [9]. Unlike 229E and OC43, SARS-CoV infection causes lower respiratory tract infection, accompanied by a cytokine storm in patients with poor outcome. Life-threatening ARDS was developed in some critically ill patients. Apart from a respiratory infection, gastrointestinal and CNS infection was also found in some patients with SARS [10,11]. FRHK4 and Vero-E6 cells played an important role in the discovery of SARS-CoV [9]. The same as chicken embryos in which other CoVs including avian infectious bronchitis virus were isolated and cultured, these cells are highly susceptible to SARS-CoV infection because they are type I interferon-defective [12]. Consistent with this, infection of STAT1^−/−^ mice with SARS-CoV resulted in a lethal outcome with a profibrotic phenotype in the lung [13]. These mice cannot clear the virus. All these highlight the importance of host antiviral response in the control of SARS-CoV infection at the cellular and organismal level.

In the post-SARS era, CoV research was brought back to the limelight and more effort was put into the search for novel HCoVs. The search was fruitful and two new HCoVs were identified in human samples positive for HCoV but not for SARS-CoV. NL63 and HKU1 were first isolated from a child suffering from bronchiolitis and a patient with pneumonia, respectively [14,15]. HKU1 is difficult to culture and can only be propagated in primary human airway epithelial cells cultured at the air–liquid interface. Similar to 229E and OC43, NL63 and HKU1 were found worldwide, causing mild respiratory diseases. Particularly, NL63 infection was associated with virus-induced croup in children [16]. All these four viruses are community-acquired HCoVs that are well adapted to humans. Only in rare cases, they might be accidentally mutated to cause more severe lower respiratory tract disease. For example, a subtype of NL63 was recently found to be associated with severe lower respiratory tract infection in China [17].

Another highly pathogenic HCoV outbreak emerged in 2012 from Saudi Arabia. A new HCoV subsequently named MERS-CoV was isolated from patients who developed acute pneumonia and renal failure [18]. Exported MERS cases were also reported outside Arabian Peninsula occasionally. One relatively big secondary outbreak with 186 confirmed cases occurred in South Korea in 2015. Up to January 2020, >2500 laboratory-confirmed case were reported with a case fatality of 34.4%. Clinical symptoms were diverse in MERS patients, ranging from asymptomatic to ARDS [19]. Acute renal injury was unique in MERS patients, but it is more commonly observed in the Middle East than in South Korea. MERS-CoV replicates well in many different types of cells and extrapulmonary tissues including the kidney and intestinal tract [20,21]. MERS-CoV is endemic in Arabian Peninsula with sporadic, but recurrent outbreaks occurring continuously since 2012.

## Animal hosts of HCoVs

The animal origin of HCoVs is supported by similarities in genome organization and phylogenetic relatedness of animal CoVs and HCoVs as well as the geographical coincidence of these viruses and plausible routes of cross-species transmission such as petting, butchering and close contact. Error-prone RNA-dependent RNA polymerase creates diversity in the CoV genome, enabling them to jump across the species barrier. However, HCoVs encode a proofreading exoribonuclease (ExoN) that plays a crucial role in RNA synthesis and replication fidelity [22]. This serves to reduce errors in RNA replication. The inactivation of ExoN causes a mutator phenotype and the resultant virus is either attenuated or inviable. In addition, other structural and non-structural genes might also contribute to the genomic diversity of CoVs by modulating polymerase and ExoN activity [23]. In addition to mutations, recombination and deletion also play an important role in host switching and adaptation. Among SARS-CoV, SARS-CoV-2, and MERS-CoV, a mutation rate as high as 0.80–2.38 × 10^−3^ nucleotide substation per base per year has been documented for SARS-CoV [24]. This is comparable to those of primate lentiviruses, including HIVs. Compared to SARS-CoV, the variabilities in SARS-CoV-2 and MERS-CoV genomes are much less dramatic. It will be of interest to clarify how this might relate to their host adaptability. In this regard, adaptive mutations in the S protein of SARS-CoV have been found during the outbreak to result in better binding with the ACE2 receptor. Cryo-EM analysis has provided structural evidence that S protein of SARS-CoV-2 binds with ACE2 with higher affinity. It will be of interest to see whether SARS-CoV-2 might be further adapted to ACE2 in the near future. Since the receptor-binding domain also contains predominant neutralizing epitopes, variations in this domain are only relevant to the development of a vaccine against SARS-CoV-2 [25].

All HCoVs have a zoonotic origin. Whereas bats are the evolutionary reservoir host of 229E, NL63, SARS-CoV, MERS-CoV, and SARS-CoV-2, parental viruses of OC43 and HKU1 have been found in rodents. Intermediate and amplifying hosts of HCoVs were also found in domestic and wild mammals. Ancestors of OC43 were identified in domestic animals such as cattle and swine. The switch of hosts from cattle or pigs to humans might have occurred in the context of a pandemic of respiratory disease recorded around 1890 in human history [26]. Similar to MERS-CoV, 229E could be acquired by humans from dromedary camels. However, the direction of this cross-species transmission remains to be determined and the possibility cannot be excluded that both humans and camels might have acquired 229E from an unidentified host including bats [27].

In an effort to identify the direct animal source of SARS-CoV, SARS-CoV-related CoVs (SARS-rCoV), which share 99.8% sequence homology at the nucleotide level with SARS-CoV, were isolated in 2003 from workers working in a live animal market where animal meats were sold and from animals in the same market [28], including Himalayan palm civets and a raccoon dog. Palm civets were once thought to be the natural host of SARS-CoV as anti-SARS-CoV antibody was detected in civets in the market. In experimental infection, civets were equally susceptible to SARS-CoV and SARS-rCoV. Infected animals displayed clinical symptoms. However, no anti-SARS-CoV antibodies were detected in any wild or farmed civets [29], raising the possibility that they are not a natural host of SARS-CoV and SARS-rCoVs. In 2005, horseshoe bats were identified as a natural host of SARS-rCoVs [30,31]. These bat SARS-rCoVs serve as the gene pool and an evolutionary origin of SARS-CoV. It is particularly noteworthy that a SARS-rCoV using the same ACE2 receptor as SARS-CoV was also found in bats [32]. Their genomes share 95% nucleotide sequence homology. Presumably, palm civets and other mammals in the market were transiently infected, and they transmitted the virus to humans. It remains to be clarified whether another stable and natural reservoir host of SARS-CoV, exactly like dromedary camels for MERS-CoV, might exist.

The genomic sequence of MERS-CoV was closely related to bat CoVs HKU4 and HKU5 [18]. Bat CoVs that are evolutionarily closer to MERS-CoV, sharing ∼75% nucleotide sequence homology and using the same DPP4 receptor, were also identified [32]. Although bats are the evolutionary reservoir host and bat CoVs serve as the gene pool of MERS-CoV, humans acquire MERS-CoV from diseased dromedary camels, but not directly from bats. These camels are the natural reservoir host of MERS-CoV. MERS-CoVs isolated from dromedaries are identical to those found in humans. Experimental infection of dromedary camels with MERS-CoV results in mild disease, shedding large quantities of the virus from the upper respiratory tract [33]. In addition, other non-camelid domestic animals in close contact with infected camels, including sheep, goats, a cow, and donkeys, are also infected by MERS-CoV [34]. These domestic animals could also pose a risk to humans and should, therefore, be included in the MERS-CoV surveillance programme.

SARS-CoV-2 was found to share 96.2% nucleotide homology with a bat CoV RaTG13 found in *Rhinolophus affinis* bats [35]. However, their receptor-binding domains in the S proteins differ significantly. Some of the earliest patients infected with SARS-CoV-2 were linked to the Huanan Seafood Wholesale Market and other live animal markets in Wuhan, Hubei, China [36]. SARS-CoV-2 was detected from the working environment of the market, supporting the existence of a live animal source. Bamboo rats in the family of *Rhizomyidae* and civets are the prime suspects of an intermediate host of SARS-CoV-2, although no concrete evidence is available. Metagenomic analysis of CoV sequences indicates that pangolins, which are a group of endangered small mammals, carry betacoronaviruses at a high rate [37], including some sharing ∼90% nucleotide homology with SARS-CoV-2. The pangolin betacoronaviruses are phylogenetically related to both SARS-CoV-2 and RaTG13. Existing evidence suggests that neither RaTG13 nor pangolin betacoronaviruses might be the immediate ancestor of SARS-CoV-2. Further investigations are required to determine whether pangolins and other animals might harbour parental viruses of SARS-CoV-2 and serve as its intermediate and amplifying host.

## Bats as a reservoir of emerging viral pathogens of humans

As the only flying mammals, bats are known as a natural reservoir of various human pathogenic viruses including but not limited to rabies virus, Nipah and Hendra viruses, Ebola virus, and influenza viruses. They can directly transmit rabies virus, Nipah and Hendra viruses, and Ebola virus to humans. Ebola virus might also be transmitted to humans indirectly through fruits contaminated by fruit bats in the African forests. Due to large geographical distribution and great diversity of bat species, a large number of bat CoVs can be created through inter-genus and inter-species transmission and recombination [38].

CoV-infected bats are asymptomatic or have mild symptoms suggesting that CoVs and bats are mutually adapted to high degrees [38]. Particularly, bats are well adapted to CoVs anatomically and physiologically. First, a high level of reactive oxygen species (ROS) generated from the high metabolic activity may suppress CoV replication in bats to a manageable level. Second, degeneration of inflammatory sensors and NF-κB signalling pathway in bats attenuates virus-induced pathology [39]. Particularly, NLRP3 inflammasome activation is defective in bats [40]. Third, constitutively active type I and III interferon production and innate immune response suppress viral replication through the persistent expression of interferon-stimulated genes [41]. It has been speculated that endogenous retroviruses in bats help to sustain interferon stimulation in bats. On the other hand, STING signalling is defective in bats and this might lead to selective repression of a subset of interferon-stimulated genes [42]. Finally, upregulation of inhibitory natural killer cell receptor NKG2/CD94 and low expression level of major histocompatibility complex class I molecules in bats may hinder natural killer cell activity [43]. All these unique features empower bats to survive CoV infection and to co-exist with a large number of bat CoVs. Moreover, a high metabolic rate in bats may provide the selection pressure for the generation of highly pathogenic virus strains. High ROS level in bats is mutagenic by affecting proofreading of CoV polymerase [38]. More pathogenic CoV strains may be generated by recombination, leading to the acquirement of novel proteins or protein features for host adaptation. Bats have an average life span of >25 years [38]. The long life span and the possible establishment of persistent virus infection in bats increase the chance for cross-species transmission of bat CoVs [38].

## Lessons from HIVs

HIVs are the most studied viruses in history and the best model to understand the interplay between virus and host antiviral defence. Tracing the origins of HIVs would provide a framework for us to understand cross-species transmission and pathogenicity of SARS-CoV-2. The comparison of SARS-CoV-2 and HIVs would reveal a common theme and the requirements for their successful species jumping. In particular, lessons learnt from HIVs are highly relevant and instructive to SARS-CoV-2 for the following reasons. First, both HIVs and SARS-CoV-2 are of zoonotic origin. Second, infection of their reservoir hosts with parental viruses of HIVs and SARS-CoV-2 results in no or mild symptoms. However, when they infect humans, much more severe symptoms are developed. Third, the similarities and differences between HIV-1 and HIV-2 resemble those between SARS-CoV and SARS-CoV-2. Finally, both HIVs and SARS-CoV-2 are plausibly derived from discrete cross-species transmission events from animals to humans. Thus, we will briefly review our current understanding of the origins of HIVs and how host anti-HIV defence has shaped the emergence of the pandemic HIV strains.

There is persuasive evidence that HIVs are derived from multiple cross-species transmissions of simian immunodeficiency viruses (SIVs) that naturally infect African non-human primates. The pandemic HIV-1 strain of group M originated from a single transmission event from a chimpanzee that harbours SIVcpz near Cameroon in Central Africa. Multiple other transmission events of SIVs from chimpanzees to humans were also detected, but their resulting HIV-1 viruses in groups N, O and P spread in humans only to a limited extent [44]. Group O was found in a few tens of thousands of people in West-Central Africa. Groups N and P were identified in 13 and 2 individuals, respectively. Likewise, appreciable spreading of HIV-2 within humans is seen only with groups A and B resulting from two cross-species transmissions of SIVsmm from sooty mangabeys in West Africa [44]. All other groups (C–H) were found only in single individuals. Thus, both HIV-1 and HIV-2 originated from one or two primate-to-human transmission events. The other transmission events were unproductive, representing incidents in which secondary and tertiary spreading was very limited.

SIVs are non-pathogenic in their natural hosts, but their transmission to a new host, such as humans for HIV-1 and HIV-2 as well as macaques for SIVmac, enable them to become highly pathogenic. HIV-1 and HIV-2 share 40–60% nucleotide sequence homology. The transmission rate of HIV-2 is lower because the viral load is generally lower in infected individuals. The natural history of HIV-2 infection is quite different from that of HIV-1. Although clinical symptoms of acquired immunodeficiency syndrome (AIDS) caused by HIV-1 and HIV-2 are indistinguishable, most people infected with HIV-2 do not progress to AIDS. One strong predictor of disease progression that distinguishes pathogenic HIV infection and non-pathogenic SIV infection is the activation of host antiviral defence including a prominent stimulation of T cells in the former but not the latter. Another possibility is that the natural hosts of SIVs might be the survivors of ancient SIV pandemics. One prediction is that HIVs and humans will eventually adapt to each other just like SIVs and their natural hosts. In this regard, AIDS might be considered an accident in which HIVs fail to adapt to humans or humans fail to adapt to HIVs. In support of this view, species-specific features in host restriction factors, such as TRIM5α and tetherin, can prevent SIV infection of humans. On the other hand, adaptive mutations and accessory genes such as Vpu, Nef, and Vif in HIVs have been found to counteract host restriction factors, which constitute the antiviral defence, in a host-specific manner. For example, a five-codon deletion in the cytoplasmic domain of human restriction factor tetherin results in the prevention of its interaction with SIVcpz Nef in humans [45]. On the side of the virus, some HIV-1 strains use their Vpu protein to degrade tetherin [45].

The origins of another pair of human retroviruses named human T lymphotropic viruses 1 and 2 (HTLV-1 and HTLV-2) are also very similar and relevant to HCoVs [46]. HTLV-1 and HTLV-2 share ∼70% nucleotide sequence homology. Whereas HTLV-1 causes a highly lethal disease named adult T-cell leukaemia and another immune-mediated disorder of the spinal cord, the related virus HTLV-2 is largely non-pathogenic and non-oncogenic. Both viruses have counterparts in non-human primates and so are HTLV-3 and HTLV-4 newly discovered in Cameroonian hunters of non-human primates [47]. At least four cross-species transmission events of HTLVs have been identified, each of which involves a different species of primates. The spreading of HTLV-3 and HTLV-4 is very limited in humans, but HTLV-1 and HTLV-2 have infected millions of people. The infection of T lymphocytes with HTLV-2 provides a good example of asymptomatic infection in humans.

HIVs and SARS-CoVs bear many similarities in terms of cross-species transmission. It is difficult to predict how the ongoing outbreak of SARS-CoV-2 might develop in the coming weeks and months. Unprecedented measures have now been taken to isolate the sources of SARS-CoV-2 infection, to block human-to-human transmission and to protect the susceptible individuals. It remains to be seen whether and to what extent secondary and tertiary spreading will be weakened and prevented by the control measures. Apparently, the intrafamily transmission of SARS-CoV-2 has not been stopped in the epicentre of Wuhan after 23 January 2020, when the city was locked down and human gathering was prohibited. It also remains to be determined what percentage of the general population in Wuhan have been or are being infected by SARS-CoV-2. These are important research questions that should be set as priority. However, as seen in HIV-1, HIV-2, HTLV-3, and HTLV-4, not every animal-to-human transmission event gives rise to a virus that is highly and sustainably transmissible within humans. The transmission of SARS-CoV-2 might be stopped due to the intrinsic characteristics of the virus, the action of human restriction factors, and human intervention measures. Another possibility is that SARS-CoV-2 becomes highly transmissible within humans just like the other four community-acquired HCoVs. Some estimates of the transmission rate expressed as reproductive number (*R*_0_) of SARS-CoV-2 fall within the range of 3–4, which is higher than that of SARS-CoV (Table 1). If that can be sustained, SARS-CoV-2 will be well adapted to humans ultimately. It will be fortunate if it also becomes less pathogenic, resembling 229E, OC43, HKU1, and NL63. Plausibly, when they initially crossed species barriers to infect humans decades or centuries ago, 229E, OC43, HKU1, and NL63 might have also caused pandemics in which humans were suffering from more severe respiratory diseases. As mentioned above, one such pandemic recorded at the end of nineteenth century has now been linked to the jumping of OC43 from cattle to humans [26].

**Table 1.** Comparison between SARS-CoV and SARS-CoV-2.

	SARS-CoV	SARS-CoV-2
Virus origin	Horseshoe bats as evolutionary reservoir host Civets as intermediate amplifying host Unknown reservoir host(s)?	*Rhinolophus affinis* bats as evolutionary reservoir host Unknown intermediate amplifying host(s)? Unknown reservoir host(s)?
Entry receptor	ACE2 as entry receptor Both human ACE2 and civet ACE2 capable of supporting SARS-CoV entry Mouse ACE2 less efficient in supporting entry of SARS-CoV when compared with human ACE2	ACE2 from humans, *Rhinolophus sinicus* bats, civets and swine as entry receptor [35] Mouse ACE2 unable to serve as entry receptor [35]
Human-to-human transmission route	Droplets in most cases Close contact with contaminated fomites Faecal–oral Aerosols uncommon but possible under special circumstances	Droplets in most cases Close contact with contaminated fomites Faecal–oral Aerosols uncommon but possible under special circumstances Higher attack rate within family clusters
Superspreading events	Superspreading events detected in Hong Kong and Beijing [8]	Superspreading events suspected as in the Diamond Princess cruise ship.
Clinical presentations	Lower respiratory infection ICU care required in ∼30% patients ARDS in ∼20% patients Gastrointestinal and CNS infection	Lower respiratory infection [36] ICU care required in 5–10% patients ARDS in 5% patients [36] Gastrointestinal infection Asymptotic carriers [51]
Case fatality	9.6% worldwide	3.4% worldwide as of 24 February 2020 (4.0% in Hubei Province, China, and 0.84% elsewhere)
Transmissibility	*R*_0 _= 2^a^	*R*_0 _= 3–4^b^
Interferon antagonists	nsp1, nsp3, nsp16, ORF3b, ORF6, M and N proteins	nsp1, nsp3, nsp16, ORF3b, ORF6, M and N proteins?
Inflammasome activators	ORF3a, ORF8b, and E proteins	ORF3a, ORF8, and E proteins?

^a^*R*_0_ is <1 for tertiary and quaternary spreading as well as in the later phase.

^b^It remains to be seen as to whether R_0_ will substantially reduce in tertiary and quaternary spreading as well as in the later phase.

## How similar and different are SARS-CoV and SARS-CoV-2?

As viruses in the same lineage, SARS-CoV and SARS-CoV-2 are very similar (Table 1), sharing 82% nucleotide sequence homology [48]. Known interferon antagonists encoded by SARS-CoV include nsp1, nsp3, nsp16, ORF3b, ORF6, M and N proteins [49]. They, respectively, share 84, 76, 93, 32, 69, 91, and 94% amino acid sequence identity with their counterparts in SARS-CoV-2. Known activators of NLRP3 inflammasome encoded by SARS-CoV include E, ORF3a, and ORF8b [50]. They, respectively, share 95, 72, and 40% amino acid identity with their counterparts in SARS-CoV-2. It is noteworthy that some accessory proteins that modulate interferon response and inflammasome activation in the two viruses varied substantially. It will be of interest to see whether the divergence might have affected the virulence and pathogenicity of SARS-CoV-2.

Comparison of the sequence and genome organization of SARS-CoV and SARS-CoV-2 reveals more similarities than differences. Overemphasizing the differences in the initial stage of the outbreak has turned out to be counterproductive and very costly in disease control. The sequence similarities predict that the patterns and modes of the interaction between SARS-CoV-2 and host antiviral defence would be similar. Indeed, they share many features during the course of infection. First, they share the same cellular receptor ACE2 [35]. Second, their transmission routes and patterns are very similar. While both are transmitted through droplets primarily, close contact is a major risk factor. The attack rate of SARS-CoV-2 within the family context is even higher than that of SARS-CoV [36,51]. The faecal–oral route for transmission of SARS-CoV-2 has been reported as in the case of SARS-CoV. More studies are required to elucidate the exact role of faecal–oral transmission in the spreading of SARS-CoV-2. Third, superspreading events have been documented for SARS-CoV [52] and are also suspected to have occurred in the transmission of SARS-CoV-2, which could explain the rapid increase in confirmed cases in many places including 691 on the Diamond Princess cruise ship as of 23 February 2020. Fourth, clinical presentations of SARS-CoV and SARS-CoV-2 infection are similar, although symptoms associated with SARS-CoV-2 infection are generally milder. Fifth, host antiviral defence plays a critical role in the course of both SARS-CoV and SARS-CoV-2 infection. For severe cases, immunopathogenesis and induction of a proinflammatory cytokine storm are the culprit. Finally, drugs tested effective for SARS-CoV have been shown to exhibit an anti-SARS-CoV-2 effect; examples include nucleotide analogue Remdesivir [53], protease inhibitors Lopinavir and Ritonavir, as well as interferon α2a. Particularly, activation of innate antiviral response by interferon α2a should have beneficial effects at least in the initial stage of infection. However, cautions should still be observed and the possibility that interferon α2a might exacerbate inflammation during the late phase of SARS-CoV-2 infection cannot be excluded. Other innate immune stimulators should also be tested for anti-SARS-CoV-2 effects in future *in vitro* and *in vivo* experiments.

## Asymptomatic carriers: fact or fiction?

An asymptomatic carrier of SARS-CoV-2 was reported in the first study of a family cluster [51]. Transmission of SARS-CoV-2 from an asymptomatic carrier to close contacts was later suggested, but this has subsequently been challenged. However, even if family members and close contacts could be infected by the index patient in the presymptomatic window period as claimed, it is still worthy of a significant concern. Presumed transmission of SARS-CoV-2 from an asymptomatic carrier to family members has recently been documented [54]. The existence of many asymptomatic carriers, presymptomatic patients, and patients with very mild symptoms posts a huge challenge to infection control, as the transmission of SARS-CoV-2 from these people to susceptible groups would be difficult to prevent. The number of people infected with SARS-CoV-2 could be underestimated. However, existing evidence suggests that the risk might probably be lower than expected. First, asymptomatic carriers are not common. In the first family cluster that was carefully studied, only one of the six family members was found to be asymptomatic or present with non-specific and mild symptoms with the typical ground-glass opacities in only one but not both lungs [51]. Second, the transmission of SARS-CoV-2 from asymptomatic carriers and presymptomatic patients could be even less common, if their viral loads are low and virus shedding is not substantial. The key questions concern how often asymptomatic and presymptomatic virus shedding might occur as well as whether their viral loads could be high.

Asymptomatic carriers of other HCoVs including 229E, OC43, NL63, and HKU1 have been well documented. Importantly, the detection rate of the virus in this group was lower and viral loads were much lower compared to patients with upper respiratory tract symptoms [55]. This is generally consistent with the notion that asymptomatic or presymptomatic shedding of SARS-CoV-2 might be less common than some estimates such as half to half. In this regard, epidemiological studies to determine the percentages of asymptomatic carriers and in selected large cohorts of subjects in Wuhan should help clarify the role of asymptomatic virus shedding in SARS-CoV-2 transmission. This analysis will also rule in or rule out our prediction that asymptomatic virus shedding exists but is uncommon. Since patients with non-specific and mild symptoms as well as asymptomatic carriers can go undetected easily, the chance that SARS-CoV-2 will be established in humans is increased. It will likely become either endemic in some regions or pandemic.

Theoretically, asymptomatic carriers might arise when host antiviral defence is either strong or decoupled. When the immune response effectively limits but could not completely block SARS-CoV-2 replication, asymptomatic shedding might occur. In this scenario, the risk of transmitting to others is relatively low because of a low viral load. Alternatively, if the immune response against SARS-CoV-2 is decoupled from viral replication as in the infection of natural primate hosts with SIVs, the viral load would be higher, posing a higher risk for person-to-person transmission. A careful quantitative analysis of the replication dynamics of SARS-CoV-2 in asymptomatic carriers over time is required to clarify the validity of the two models.

## Evolving to be less pathogenic: is there a trend?

CoVs are believed to have existed for >6000 years. Molecular clock analysis enables us to deduce the time of emergence or cross-species transmission of some HCoVs. Surprisingly, the highly pathogenic SARS-CoV and MERS-CoV are thought to emerge in the last 30 years [56]. Although NL63 and HKU1 of low pathogenicity were isolated in the post-SARS era, their time of emergence was earlier than the other two highly pathogenic viruses. Whereas NL63 emerged >500 years ago [57], the origin of HKU1 dated back to the 1950s. In other words, SARS-CoV and MERS-CoV represent newcomers that are striving to adapt to humans, whereas other community-acquired HCoVs including NL63 and HKU1 are better adapted. The results of molecular dating of HCoVs suggest that attenuation might be favoured during evolution when host adaptation takes place.

To shed further light on this trend, a comparative analysis of the innate immunomodulatory activity of viral proteins encoded by different HCoVs would be helpful. In the first place, type I interferon antagonism of these proteins might be compared. The use of interferon to treat HCoV-infected patients was tested as early as the 1960s, when various volunteer experiments were performed to investigate how humans combat HCoV infection. Administration of interferon ameliorated the severity of symptoms in 229E-challenged volunteers [58], suggesting that interferon confers protection against 229E infection. Indeed, 229E potently induces type I interferon and its replication is susceptible to inhibition by type I IFN [59], suggesting that interferon serves as a key component of host antiviral defence. Since interferon effectively suppresses the early phase of viral replication, the suppression of interferon production and signalling by highly pathogenic HCoVs can exacerbate disease progression. Thus, highly pathogenic HCoVs adopt various countermeasures to suppress host interferon production and signalling as reviewed elsewhere [49].

Host innate immune response is the first-line defence triggered by type I interferon. Type I interferon production is activated through the detection of replicating viral RNA by cytoplasmic RNA sensors RIG-I and MDA5. Oligomerization of adaptor protein MAVS is induced by the activation of RNA sensor, leading to the formation of TRAF3-TANK-TBK1/IKKϵ complex, which phosphorylates transcription factor IRF3 and drives type I IFN transcription [49]. Highly pathogenic HCoVs often encode viral proteins with a higher capability to antagonize RNA-induced type I interferon production through perturbation of RNA sensing. For one example, double-stranded RNA (dsRNA)-binding domain of MERS-CoV ORF4a is responsible for the suppression of Sendai virus- or poly (I:C)-induced type I interferon production [60]. The gain of dsRNA-binding ability is observed in bat CoV HKU5 but not HKU4, suggesting that the functional gain of MERS-CoV ORF4a and HKU5 ORF4a might be acquired at a later stage in evolution [60]. For another example, only M proteins from highly pathogenic CoVs, SARS-CoV and MERS-CoV, were reported to potently suppress type I interferon production [61,62], suggesting that the loss of this activity might have taken place during viral evolution, leading to attenuation.

Other than suppressing type I interferon induction, both alphacoronaviruses and betacoronaviruses can use nsp1 to degrade host mRNA transcripts, resulting in the suppression of host interferon response, although sequence homology between nsp1 proteins of the two genera remains low. A conserved domain is present in nsp1 of alphacoronaviruses and it is responsible for the shut-down of host gene expression. The deletion of 91–95 amino acids in 229E and NL63 nsp1 partially restores host gene expression as shown by luciferase reporter assay [63]. The interferon-modulating activity of nsp1 shows some variations within the genus of *Betacoronavirus*. Whereas nsp1 proteins encoded by SARS-CoV and bat CoV Rm1 potently suppress the induction of type I interferon, counterparts in bat CoV 133 and bat CoV HKU9-1 are relatively weak interferon suppressors [64]. Again, some degree of conservation in interferon antagonism of nsp1 is seen among different CoVs.

Apart from preventing interferon production, CoV proteins have also evolved to suppress interferon effector signalling. One of the best-characterized examples is the oligoadenylate synthetase (OAS)-RNase L pathway. Upon activation by interferon in response to the sensing of dsRNA, transcription and expression of OAS are induced to catalyse 2′,5′-oligoadenylate (2′-5′A) synthesis [65]. 2′-5′A serves as a second messenger, which activates RNase L and limits viral replication through the cleavage of cellular and viral single-stranded RNA [65]. The cleaved RNA fragments, in turn, prime RNA sensors to amplify interferon production in infected cells. To counter RNase L activity, some CoVs have evolved phosphodiesterases which cleave 2′-5′A [66]. The first CoV phosphodiesterase identified is NS2a in mouse hepatitis virus. From protein sequence alignment, multiple CoVs including NS2a from OC43 and a bat CoV, as well as ORF4b encoded by MERS-CoV, are very similar to NS2a of mouse hepatitis virus. All these viral proteins preserve a phosphodiesterase activity [67]. This suggests that some CoVs have developed an important enzymatic activity through convergence and divergence. Hence, although most HCoVs retain one or another strategy to counter host antiviral defence, the highly pathogenic CoVs are particularly powerful in the suppression of host immunity. These capabilities might be weakened or lost when they adapt to humans.

Although the exact mechanisms by which SARS-CoV-2 might counteract host antiviral defence remain to be elucidated, this emerging virus is thought to be less pathogenic than SARS-CoV. Our prediction is that the IFN antagonism of SARS-CoV-2 and its ability to suppress other pathways of innate antiviral signalling might fall between those of SARS-CoV and the community-acquired HCoVs. Plausibly, the weakened IFN antagonism of SARS-CoV-2 compared to that of SARS-CoV might lead to more robust host antiviral defence, attenuated viral replication, and lower pathogenicity.

## To kill or not to kill?

Apart from the potent suppression of interferon-mediated antiviral response, highly pathogenic HCoVs often induce massive cell death and cytopathy. Cell death is a double-edged sword that can play both antiviral and proviral roles during viral infection [68]. On the one hand, it is part of the host antiviral defence that provides a dead end to viral replication and infection, often at the price of pathological changes including inflammation [69]. On the other hand, dying and dead cells release a large number of virions, facilitating viral dissemination [70].

**Figure 2.**
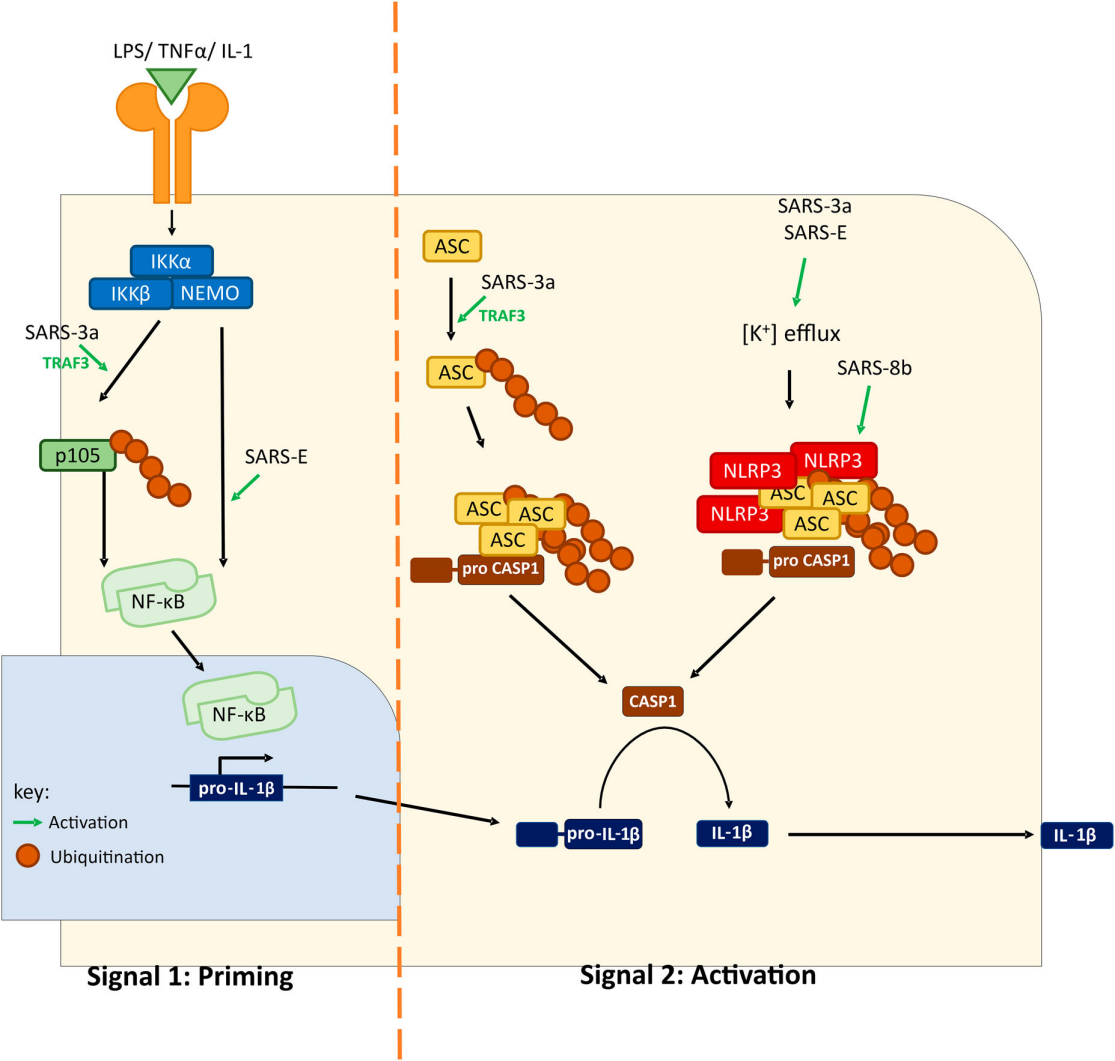
A working model of SARS-CoV-induced inflammasome activation. SARS-CoV can activate both signal 1 (priming) and signal 2 (activation). Upregulation of pro-IL-1β transcription is achieved by NF-κB activation. Two mechanisms of IL-1β maturation have been proposed. In the first model, potassium ion efflux is promoted by ORF3a and E proteins, leading to NLRP3 inflammasome assembly. Alternatively, ORF3a promotes ASC ubiquitination and consequent assembly of inflammasome. ORG8b interacts with and activates NLRP3. Activation of inflammasome leads to proteolytic cleavage of pro-caspase 1 and pro-IL-1β. ASC, apoptosis-associated speck-like protein containing a CARD. CASP1, caspase 1. IKK, IκB kinase. IL-1, interleukin-1. LPS, lipopolysaccharides. NLRP3, NACHT, LRR, and PYD domains-containing protein 3. NEMO, NF-κB essential modulator. TNF-α, tumour necrosis factor α.

One form of cell death known as pyroptosis is one of the results of a proinflammatory cytokine storm, which drives at least in part the high pathogenicity of SARS-CoV and MERS-CoV. Poor outcomes of patients with SARS and MERS are often associated with exceedingly high levels of proinflammatory cytokines in the lower respiratory tract and other tissues [71]. The upregulation of inflammatory cytokines, including interleukin (IL) 1β, was observed in SARS-CoV-infected monocyte-derived human dendritic cells and tissue models [72]. Maturation of IL-1β is generated through proteolytic cleavage of pro-IL-1β by caspase 1, the activation of which requires the formation of a multiprotein complex termed inflammasome. When danger signals are sensed in the cells, NLRP3 is activated to recruit ASC and facilitate its oligomerization. For full activation of inflammasome activity, two signals that, respectively, stimulate pro-IL-1β transcription (signal 1) and cleave pro-IL-1β (signal 2) are required. Recombinant SARS-CoVs with either deletion of ORF3a or defective ion channel activity of E protein are compromised in the activation of IL-1β maturation and secretion [73,74]. Mechanistically, both ORF3a and E can activate both signals. Both stimulates NF-κB activation, resulting in the activation of pro-IL-1β transcription [73,75]. In particular, ORF3a achieves this through TRAF3-dependent ubiquitination of p105 [73]. For the activation of the second signal, ion channel activity of SARS-CoV E protein promotes assembly of NLRP3 inflammasome [76]. Different mechanisms have been suggested for SARS-CoV ORF3a-mediated inflammasome activation. We demonstrated that SARS-CoV ORF3a promotes NLRP3 inflammasome assembly through TRAF3-dependent K63 ubiquitination of ASC [73]. Alternatively, SARS-CoV ORF3a might provide a potassium flux through its ion channel domain to activate NEK7-dependent NLRP3 inflammasome [77]. Further investigations are required to resolve the discrepancies (Figure 2). Nevertheless, enhanced secretion of IL-1β mediated by SARS-CoV E and ORF3a proteins might contribute to the induction of proinflammatory cytokine storm since IL-1β further promotes the expression of other proinflammatory cytokines such as tumour necrosis factor α and IL-6 [74,75]. Thus, small-molecule inhibitors of NLRP3 inflammasomes such as MCC950 and INF58 might prove useful in the treatment of COVID-19. This merits further preclinical and clinical studies.

Apart from cytokine storm and pyroptosis observed in highly pathogenic HCoVs, other cell death programmes such as apoptosis and necrosis might also contribute to pathogenesis. Apoptosis was detected in various HCoV-infected samples derived from not only the respiratory tract, but also extrapulmonary sites. Autopsy studies on SARS casualties revealed massive apoptosis in multiple organs including the liver and thyroid gland [78]. MERS-CoV induces apoptosis in the kidney, lung, and primary T lymphocytes, plausibly through induction of Smad7 and FGF2 [21]. To delineate how CoVs might activate apoptosis, pathway analysis was performed with CoV proteins. ORF3a, ORF3b, ORF7a, ORF8a, ORF9b, and E proteins of SARS-CoV are pro-apoptotic [79]. SARS-CoV ORF7a protein activates the intrinsic pathway of apoptosis through an interaction with anti-apoptotic protein Bcl-X_L_ in the endoplasmic reticulum, thereby sequestering a key suppressor of apoptosis. MERS-CoV infection activates both intrinsic and extrinsic pathways of apoptosis, exacerbating viral pathogenesis [21]. Other than apoptosis, SARS-CoV induces RIP3-mediated necrosis through induction of ORF3a oligomerization [80]. Generally, highly pathogenic HCoVs are capable of activating different cell death programmes more efficiently. In this regard, it will be of great interest to see whether and how the lower pathogenicity and higher human-to-human transmissibility of SARS-CoV-2 might be linked to its abilities to modulate inflammasome activation and cell death programmes selectively.

## Concluding remarks and future perspectives

The outbreak of SARS-CoV-2 provides an unprecedented opportunity for us to keep track of a zoonotic CoV that has just crossed the species barrier to infect humans. Whether the transmission of SARS-CoV-2 within humans will come to a dead end depends primarily on whether the virus has acquired the ability to transmit from person-to-person efficiently and sustainably. In the cases of MERS-CoV and SARS-CoV, secondary and tertiary spreading becomes weakened, giving the opportunity for quarantine and other measures of human intervention to take effect so that human-to-human transmission cannot be sustained. However, if transmissibility of SARS-CoV-2 is comparable to that of the other four community-acquired HCoVs and influenza viruses, we should prepare well for the arrival of another community-acquired HCoV. In this regard, it is crucial to determine the infection rate in the epicentre of Wuhan by RT–PCR and serology. The real ratios of asymptomatic carriers and patients with mild symptoms as well as the transmission rates in secondary, tertiary, and quaternary spreading are also pivotal. If the attack rate is sufficiently high, it will be tremendously challenging to contain the spreading before herd immunity develops.

The virulence and pathogenicity of SARS-CoV-2 seem to lie between those of SARS-CoV and community-acquired HCoVs. If SARS-CoV-2 becomes more attenuated as it adapts well in humans and increases its person-to-person transmissibility as anticipated, similar strategies for prevention and control of CoVs and influenza viruses might be adopted. To reduce morbidity and mortality caused by SARS-CoV-2, vaccines could be developed. If quarantine cannot contain the spreading and if it is necessary, vaccination will provide the second opportunity to eradicate SARS-CoV-2 from humans.

The interplay between SARS-CoV-2 and host antiviral defence is at the core of viral pathogenesis. It also determines the infection outcome and might explain the existence and risk of asymptomatic carriers. SARS-CoV-2 is very similar to SARS-CoV in many aspects. Lessons from other human pathogenic viruses including SARS-CoV, community-acquired HCoVs, influenza viruses, and HIVs are very enlightening and helpful. However, SARS-CoV-2 is also a novel human pathogen that may interact with host antiviral defence in a unique manner. Basic research in the field of SARS-CoV-2-host interaction holds the key to many important questions in disease control and prevention. Many important questions concerning the identity and mechanisms of interferon antagonists encoded by SARS-CoV-2 will be answered in the months and years to come. Particularly, comparative analyses of SARS-CoV-2 and SARS-CoV will advance the understanding of SARS-CoV-2 pathogenesis. The new knowledge gained will guide the development of vaccines and anti-SARS-CoV-2 therapeutics.
